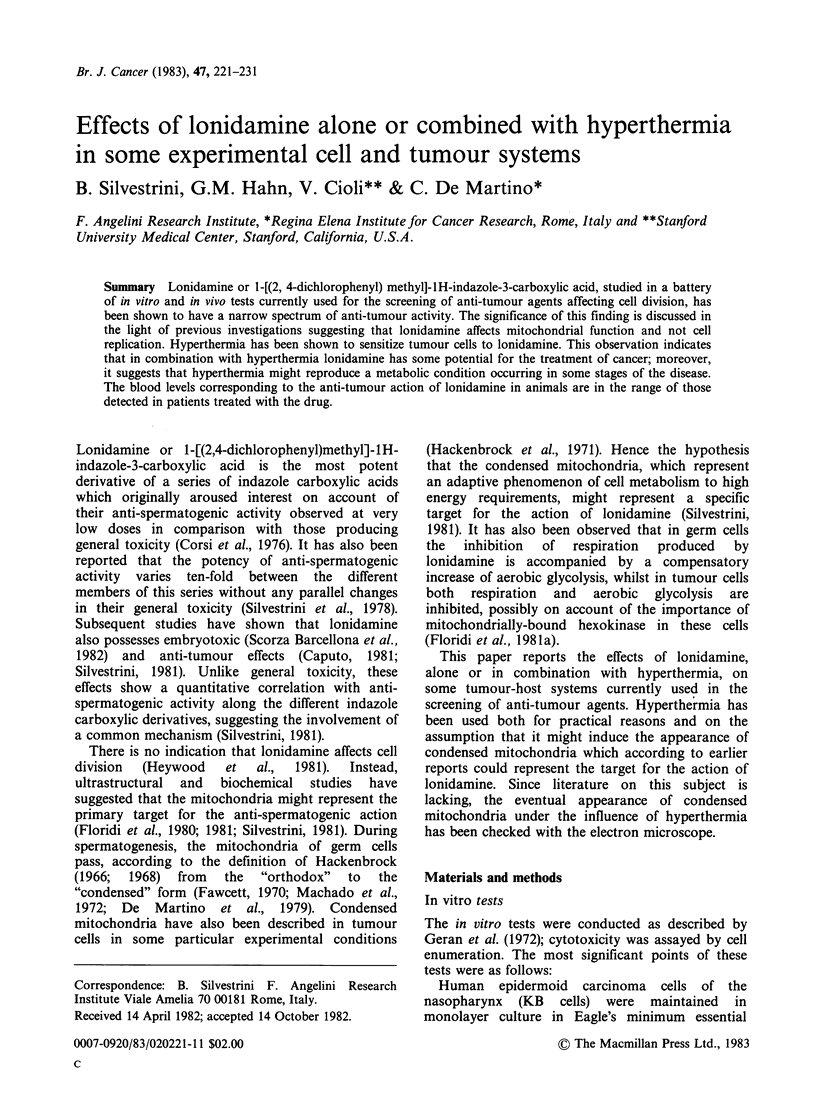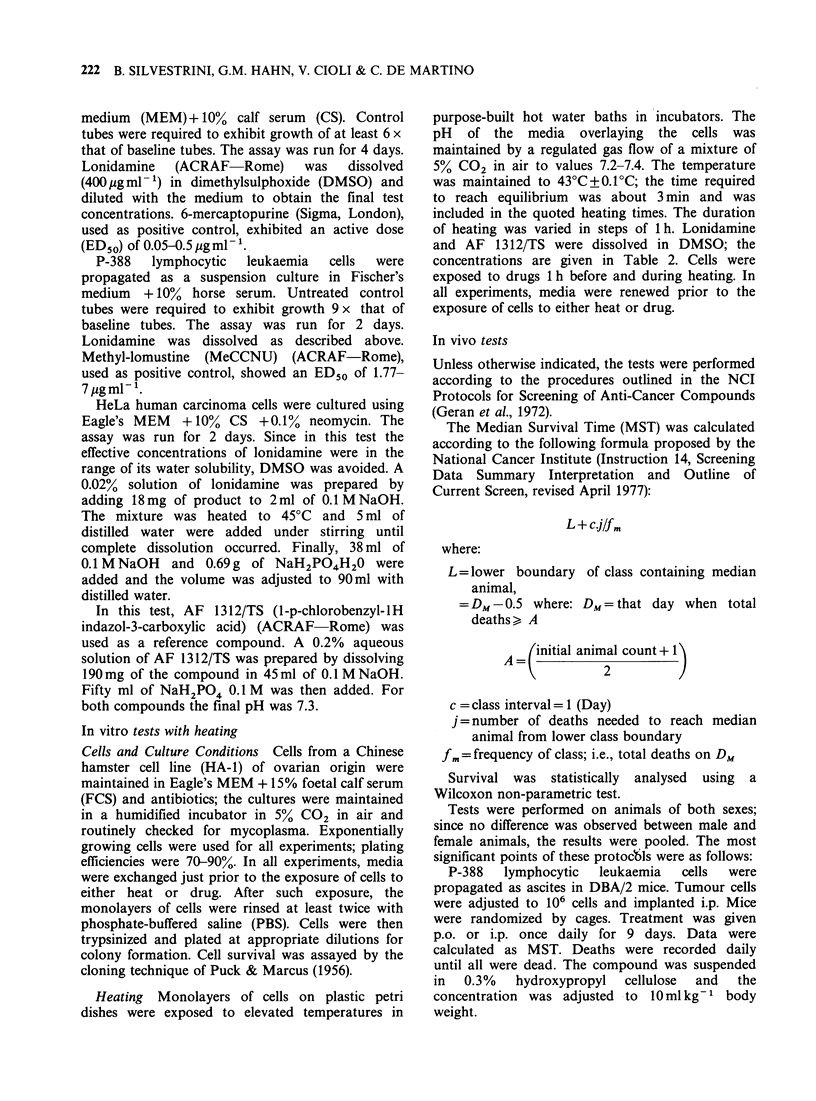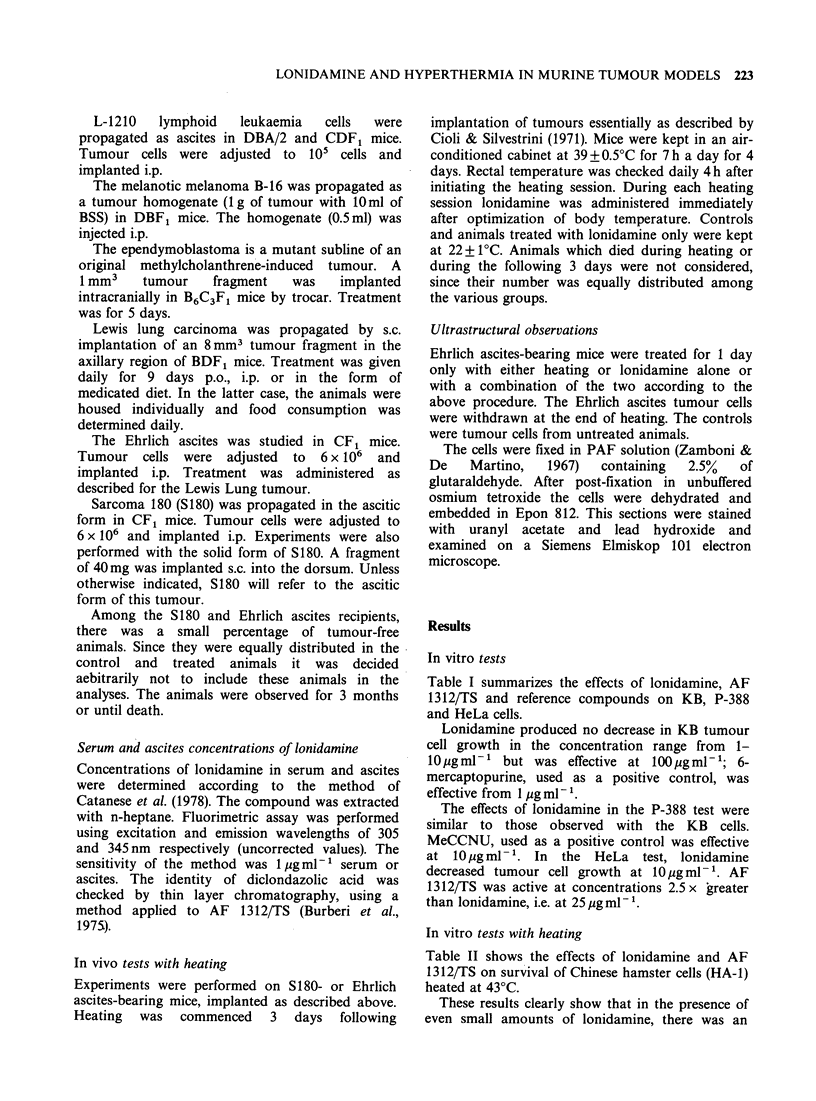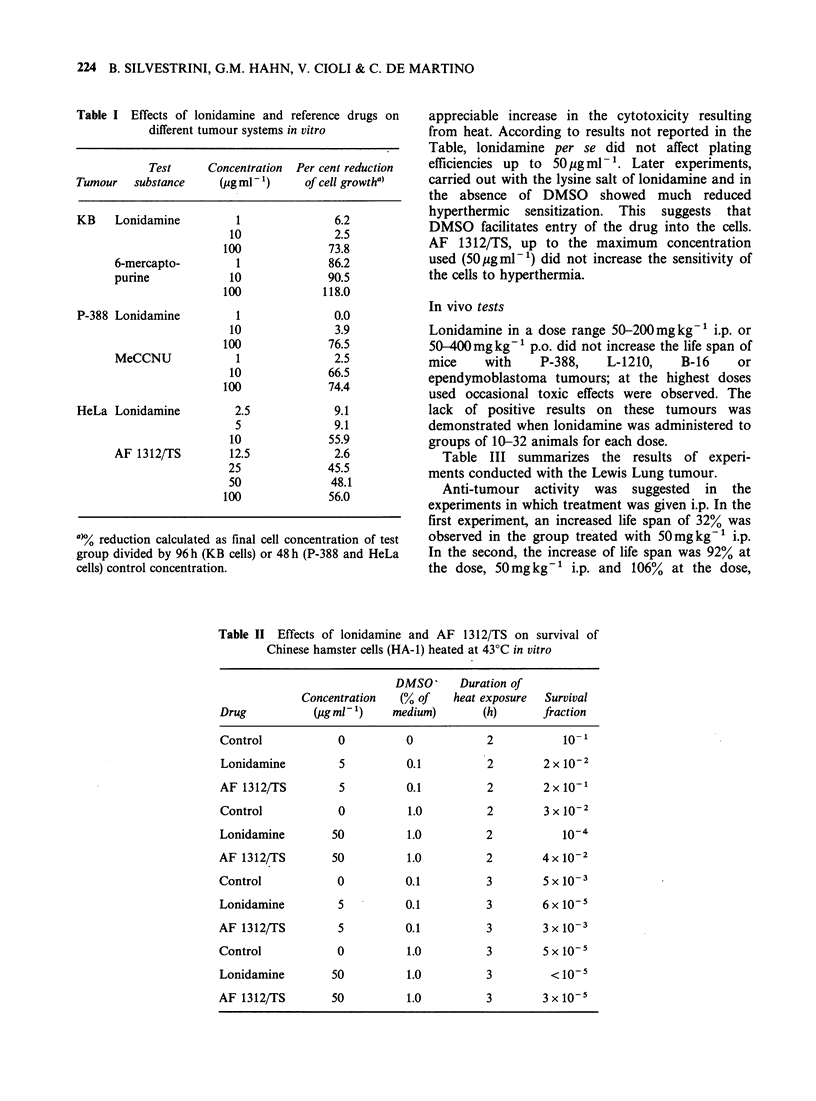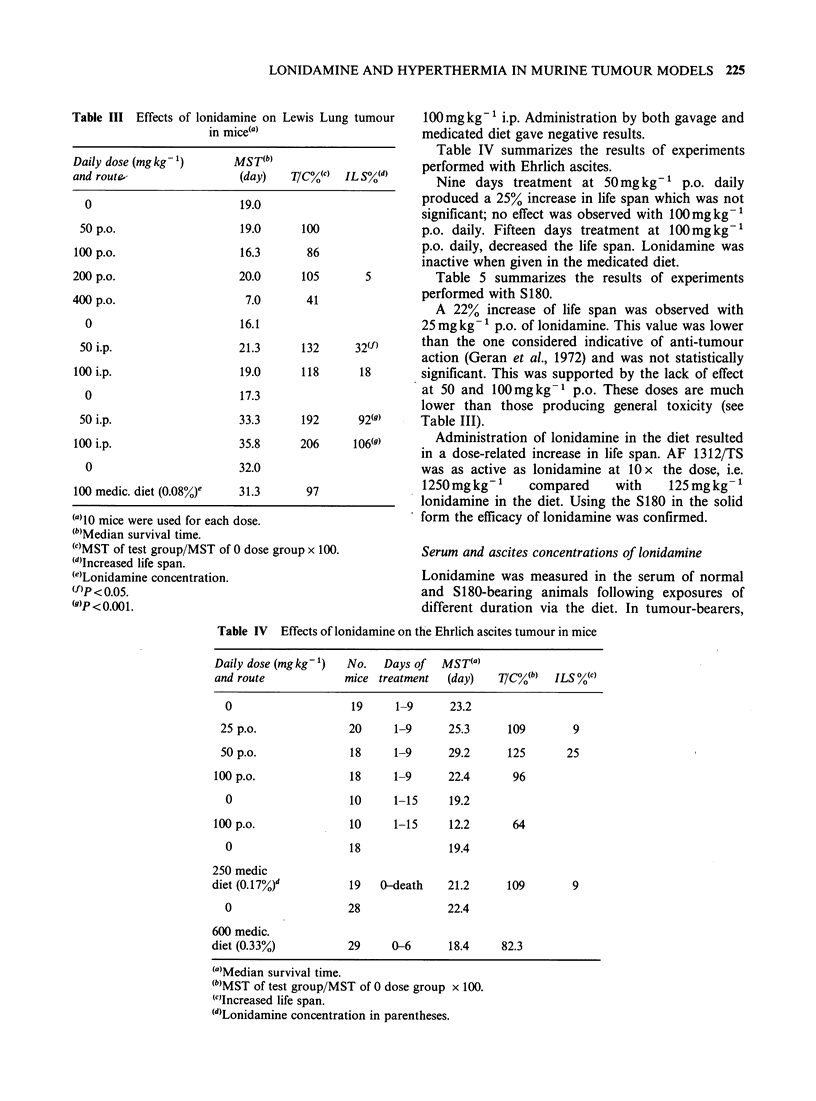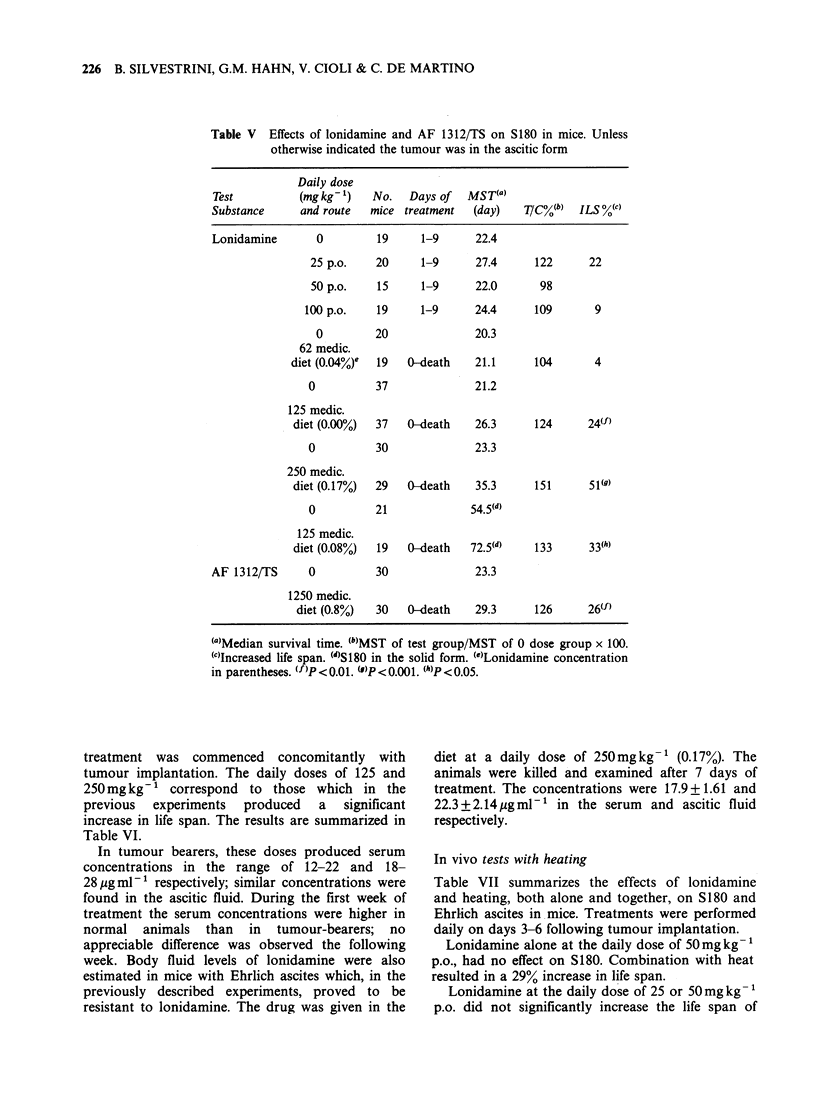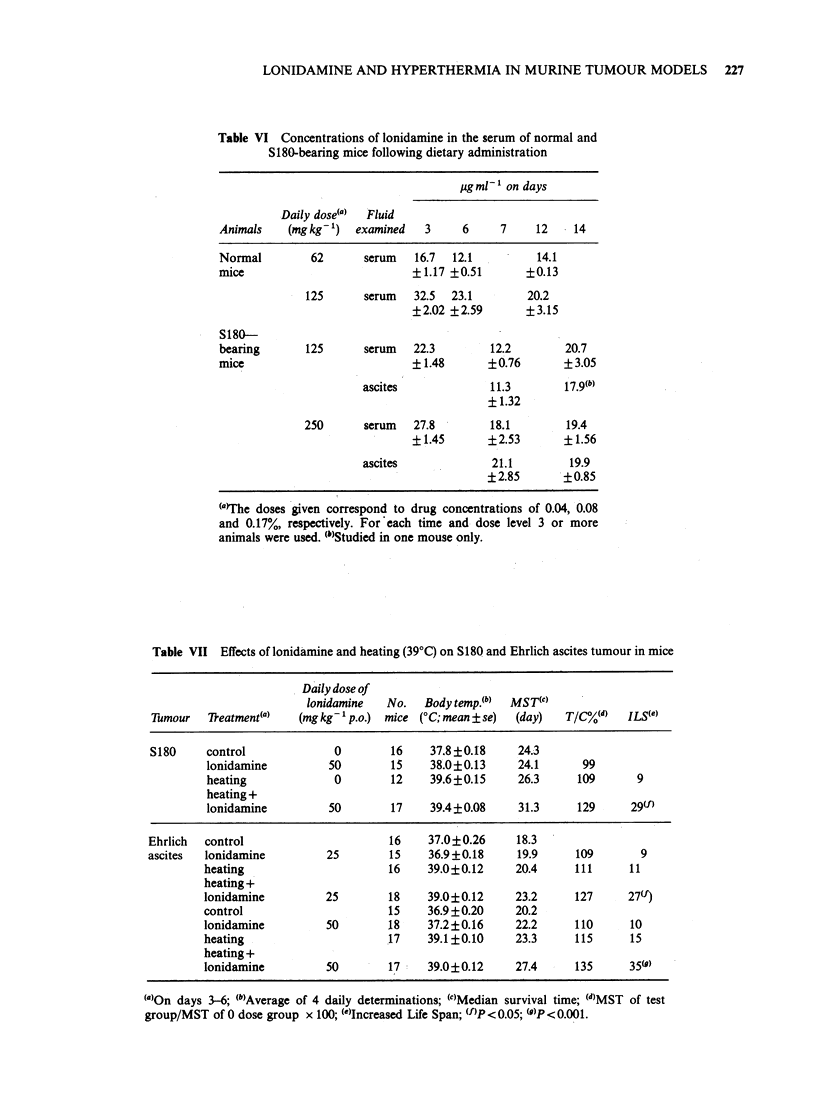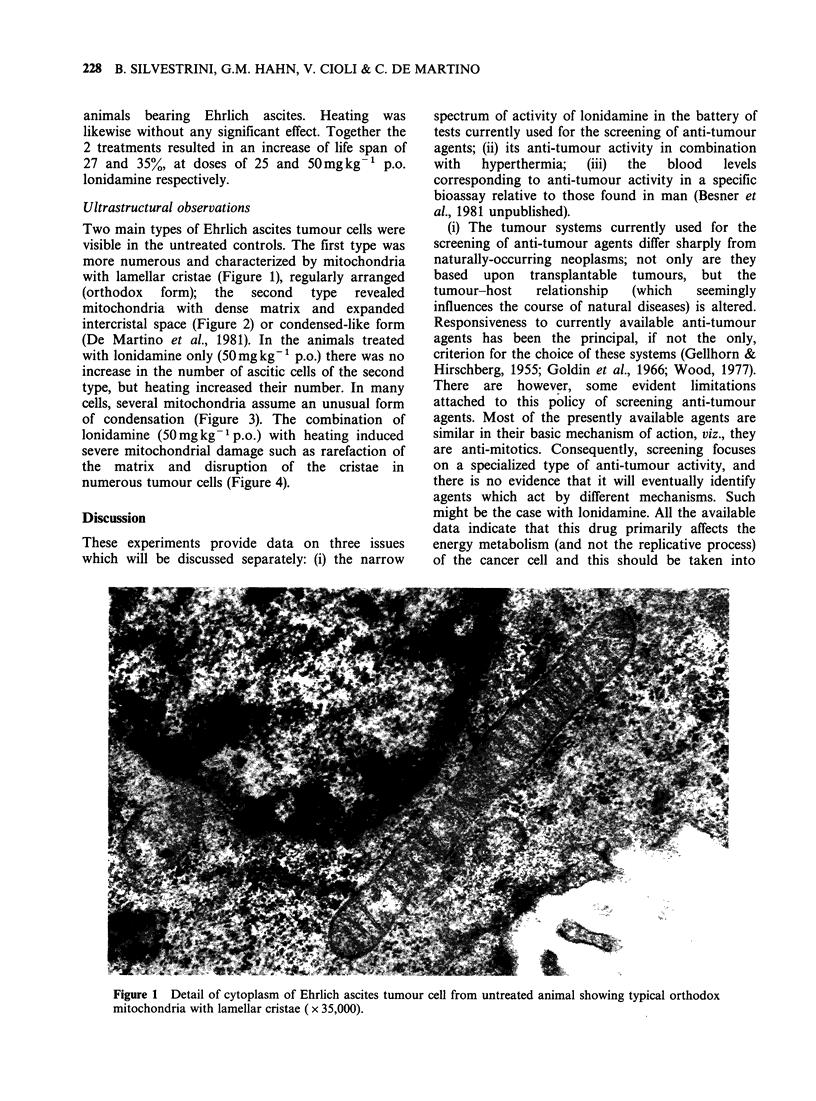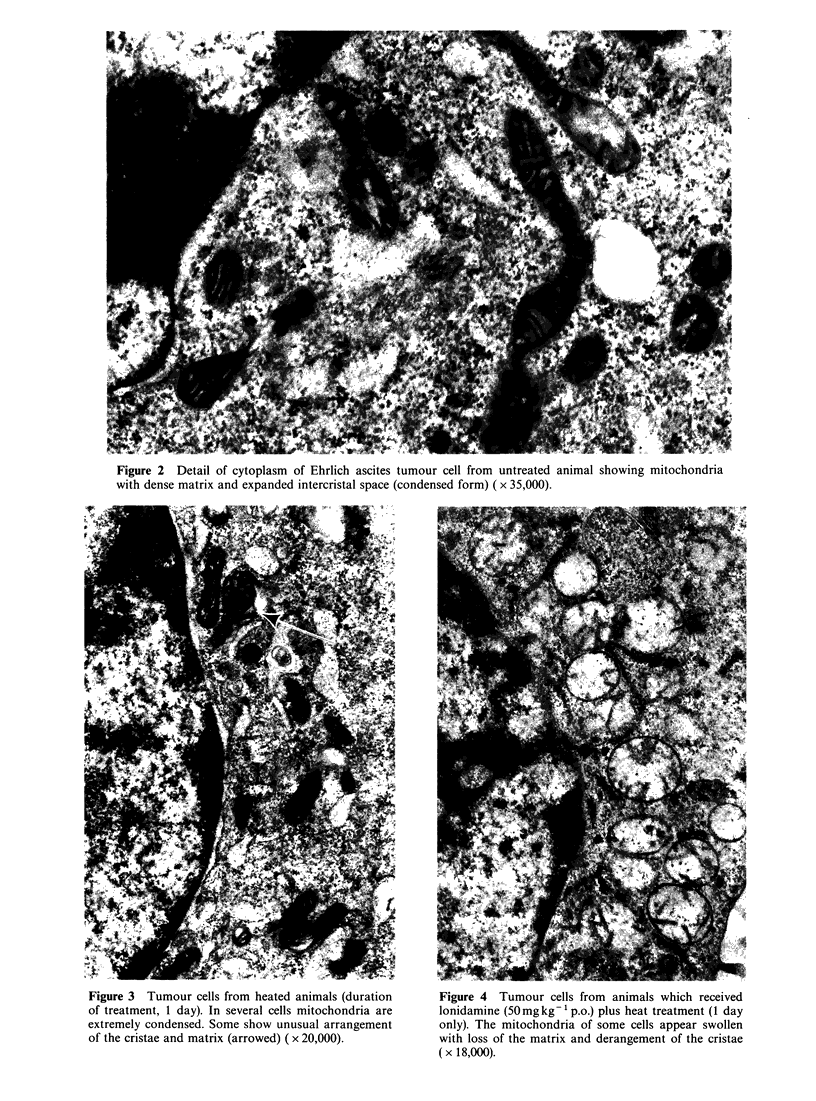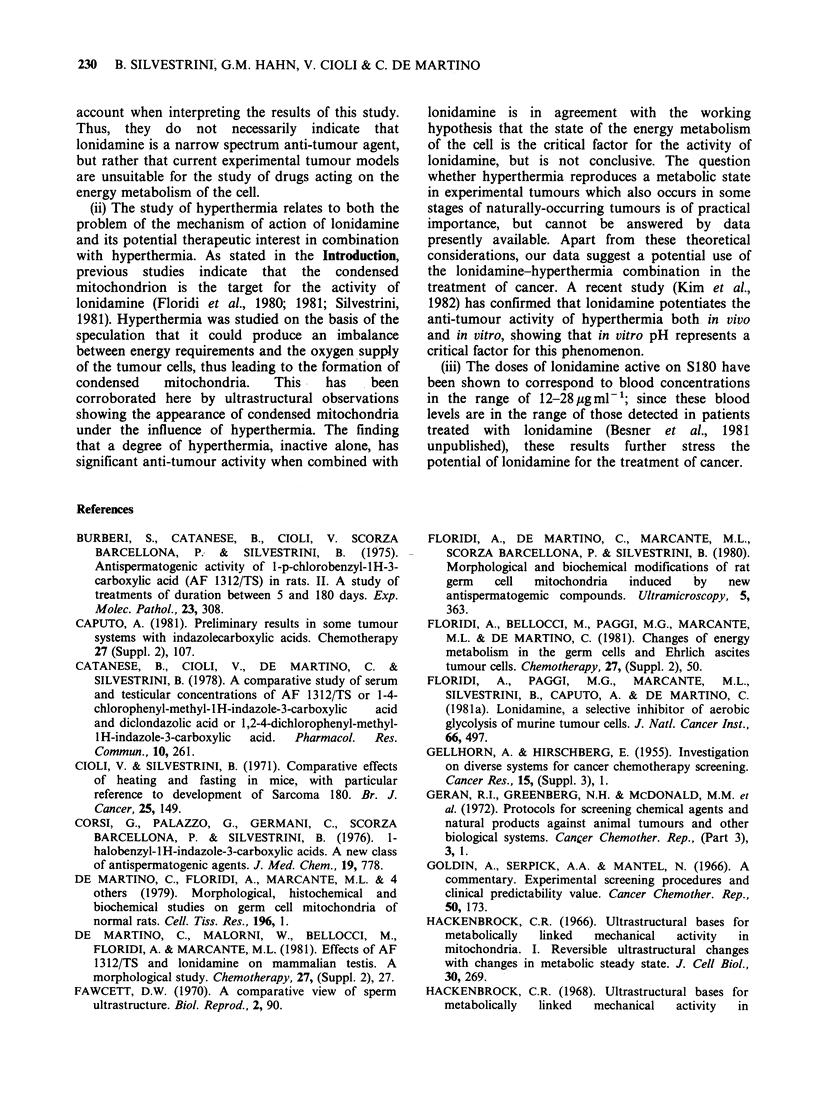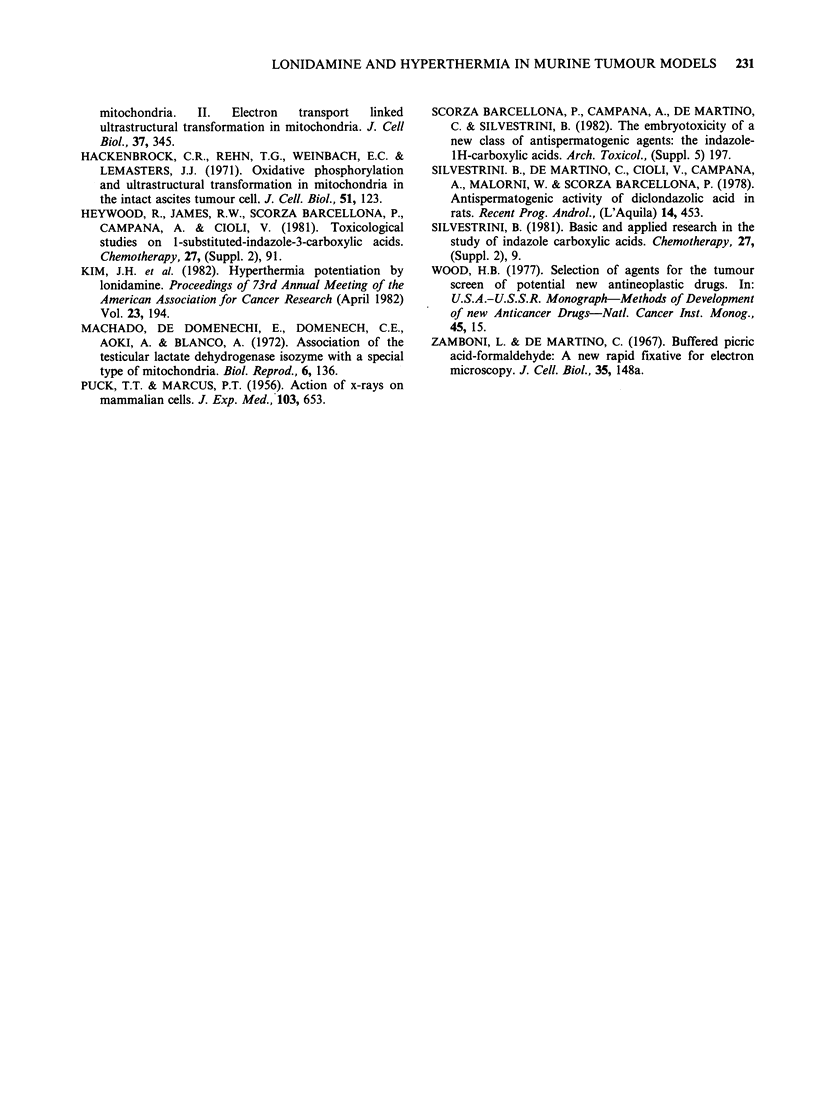# Effects of lonidamine alone or combined with hyperthermia in some experimental cell and tumour systems.

**DOI:** 10.1038/bjc.1983.30

**Published:** 1983-02

**Authors:** B. Silvestrini, G. M. Hahn, V. Cioli, C. De Martino

## Abstract

**Images:**


*Br. J. Cancer* (1983), **47**, 221–231

### Effects of lonidamine alone or combined with hyperthermia in some experimental cell and tumour systems

B. Silvestrini, G.M. Hahn, V. Cioli** & C. De Martino*

*F. Angelini Research Institute, *Regina Elena Institute for Cancer Research, Rome, Italy and **Stanford University Medical Center, Stanford, California, U.S.A.*

**Summary** Lonidamine or 1-[(2, 4-dichlorophenyl) methyl]-1H-indazole-3-carboxylic acid, studied in a battery of *in vitro* and *in vivo* tests currently used for the screening of anti-tumour agents affecting cell division, has been shown to have a narrow spectrum of anti-tumour activity. The significance of this finding is discussed in the light of previous investigations suggesting that lonidamine affects mitochondrial function and not cell replication. Hyperthermia has been shown to sensitize tumour cells to lonidamine. This observation indicates that in combination with hyperthermia lonidamine has some potential for the treatment of cancer; moreover, it suggests that hyperthermia might reproduce a metabolic condition occurring in some stages of the disease. The blood levels corresponding to the anti-tumour action of lonidamine in animals are in the range of those detected in patients treated with the drug.

Lonidamine or 1-[(2,4-dichlorophenyl)methyl]-1H-indazole-3-carboxylic acid is the most potent derivative of a series of indazole carboxylic acids which originally aroused interest on account of their anti-spermatogenic activity observed at very low doses in comparison with those producing general toxicity (Corsi *et al.*, 1976). It has also been reported that the potency of anti-spermatogenic activity varies ten-fold between the different members of this series without any parallel changes in their general toxicity (Silvestrini *et al.*, 1978). Subsequent studies have shown that lonidamine also possesses embryotoxic (Scorza Barcellona *et al.*, 1982) and anti-tumour effects (Caputo, 1981; Silvestrini, 1981). Unlike general toxicity, these effects show a quantitative correlation with anti-spermatogenic activity along the different indazole carboxylic derivatives, suggesting the involvement of a common mechanism (Silvestrini, 1981).

There is no indication that lonidamine affects cell division (Heywood *et al.*, 1981). Instead, ultrastructural and biochemical studies have suggested that the mitochondria might represent the primary target for the anti-spermatogenic action (Floridi *et al.*, 1980; 1981; Silvestrini, 1981). During spermatogenesis, the mitochondria of germ cells pass, according to the definition of Hackenbrock (1966; 1968) from the "orthodox" to the "condensed" form (Fawcett, 1970; Machado *et al.*, 1972; De Martino *et al.*, 1979). Condensed mitochondria have also been described in tumour cells in some particular experimental conditions (Hackenbrock *et al.*, 1971). Hence the hypothesis that the condensed mitochondria, which represent an adaptive phenomenon of cell metabolism to high energy requirements, might represent a specific target for the action of lonidamine (Silvestrini, 1981). It has also been observed that in germ cells the inhibition of respiration produced by lonidamine is accompanied by a compensatory increase of aerobic glycolysis, whilst in tumour cells both respiration and aerobic glycolysis are inhibited, possibly on account of the importance of mitochondrially-bound hexokinase in these cells (Floridi *et al.*, 1981a).

This paper reports the effects of lonidamine, alone or in combination with hyperthermia, on some tumour-host systems currently used in the screening of anti-tumour agents. Hyperthermia has been used both for practical reasons and on the assumption that it might induce the appearance of condensed mitochondria which according to earlier reports could represent the target for the action of lonidamine. Since literature on this subject is lacking, the eventual appearance of condensed mitochondria under the influence of hyperthermia has been checked with the electron microscope.

### Materials and methods

### In vitro *tests*

The *in vitro* tests were conducted as described by Geran *et al.* (1972); cytotoxicity was assayed by cell enumeration. The most significant points of these tests were as follows:

Human epidermoid carcinoma cells of the nasopharynx (KB cells) were maintained in monolayer culture in Eagle's minimum essential

Correspondence: B. Silvestrini F. Angelini Research Institute Viale Amelia 70 00181 Rome, Italy.
Received 14 April 1982; accepted 14 October 1982.

0007-0920/83/020221-11 $02.00
© The Macmillan Press Ltd., 1983

medium (MEM) + 10% calf serum (CS). Control tubes were required to exhibit growth of at least 6× that of baseline tubes. The assay was run for 4 days. Lonidamine (ACRAF—Rome) was dissolved ($400\,\mu g\,ml^{-1}$) in dimethylsulphoxide (DMSO) and diluted with the medium to obtain the final test concentrations. 6-mercaptopurine (Sigma, London), used as positive control, exhibited an active dose ($ED_{50}$) of $0.05$–$0.5\,\mu g\,ml^{-1}$.

P-388 lymphocytic leukaemia cells were propagated as a suspension culture in Fischer's medium +10% horse serum. Untreated control tubes were required to exhibit growth 9× that of baseline tubes. The assay was run for 2 days. Lonidamine was dissolved as described above. Methyl-lomustine (MeCCNU) (ACRAF—Rome), used as positive control, showed an $ED_{50}$ of 1.77–$7\,\mu g\,ml^{-1}$.

HeLa human carcinoma cells were cultured using Eagle's MEM +10% CS +0.1% neomycin. The assay was run for 2 days. Since in this test the effective concentrations of lonidamine were in the range of its water solubility, DMSO was avoided. A 0.02% solution of lonidamine was prepared by adding 18 mg of product to 2 ml of 0.1 M NaOH. The mixture was heated to 45°C and 5 ml of distilled water were added under stirring until complete dissolution occurred. Finally, 38 ml of 0.1 M NaOH and 0.69 g of $NaH_2PO_4H_20$ were added and the volume was adjusted to 90 ml with distilled water.

In this test, AF 1312/TS (1-p-chlorobenzyl-1H indazol-3-carboxylic acid) (ACRAF—Rome) was used as a reference compound. A 0.2% aqueous solution of AF 1312/TS was prepared by dissolving 190 mg of the compound in 45 ml of 0.1 M NaOH. Fifty ml of $NaH_2PO_4$ 0.1 M was then added. For both compounds the final pH was 7.3.

In vitro *tests with heating*

*Cells and Culture Conditions* Cells from a Chinese hamster cell line (HA-1) of ovarian origin were maintained in Eagle's MEM + 15% foetal calf serum (FCS) and antibiotics; the cultures were maintained in a humidified incubator in 5% $CO_2$ in air and routinely checked for mycoplasma. Exponentially growing cells were used for all experiments; plating efficiencies were 70–90%. In all experiments, media were exchanged just prior to the exposure of cells to either heat or drug. After such exposure, the monolayers of cells were rinsed at least twice with phosphate-buffered saline (PBS). Cells were then trypsinized and plated at appropriate dilutions for colony formation. Cell survival was assayed by the cloning technique of Puck & Marcus (1956).

*Heating* Monolayers of cells on plastic petri dishes were exposed to elevated temperatures in purpose-built hot water baths in incubators. The pH of the media overlaying the cells was maintained by a regulated gas flow of a mixture of 5% $CO_2$ in air to values 7.2–7.4. The temperature was maintained to 43°C ± 0.1°C; the time required to reach equilibrium was about 3 min and was included in the quoted heating times. The duration of heating was varied in steps of 1 h. Lonidamine and AF 1312/TS were dissolved in DMSO; the concentrations are given in Table 2. Cells were exposed to drugs 1 h before and during heating. In all experiments, media were renewed prior to the exposure of cells to either heat or drug.

In vivo *tests*

Unless otherwise indicated, the tests were performed according to the procedures outlined in the NCI Protocols for Screening of Anti-Cancer Compounds (Geran *et al.*, 1972).

The Median Survival Time (MST) was calculated according to the following formula proposed by the National Cancer Institute (Instruction 14, Screening Data Summary Interpretation and Outline of Current Screen, revised April 1977):

$$L + c.j/f_m$$

where:

$L$ = lower boundary of class containing median animal,
= $D_M - 0.5$ where: $D_M$ = that day when total deaths $\geqslant A$

$$A = \left(\frac{\text{initial animal count} + 1}{2}\right)$$

$c$ = class interval = 1 (Day)
$j$ = number of deaths needed to reach median animal from lower class boundary
$f_m$ = frequency of class; i.e., total deaths on $D_M$

Survival was statistically analysed using a Wilcoxon non-parametric test.

Tests were performed on animals of both sexes; since no difference was observed between male and female animals, the results were pooled. The most significant points of these protocols were as follows:

P-388 lymphocytic leukaemia cells were propagated as ascites in DBA/2 mice. Tumour cells were adjusted to $10^6$ cells and implanted i.p. Mice were randomized by cages. Treatment was given p.o. or i.p. once daily for 9 days. Data were calculated as MST. Deaths were recorded daily until all were dead. The compound was suspended in 0.3% hydroxypropyl cellulose and the concentration was adjusted to $10\,ml\,kg^{-1}$ body weight.

L-1210 lymphoid leukaemia cells were propagated as ascites in DBA/2 and $CDF_1$ mice. Tumour cells were adjusted to $10^5$ cells and implanted i.p.

The melanotic melanoma B-16 was propagated as a tumour homogenate (1 g of tumour with 10 ml of BSS) in $DBF_1$ mice. The homogenate (0.5 ml) was injected i.p.

The ependymoblastoma is a mutant subline of an original methylcholanthrene-induced tumour. A 1 $mm^3$ tumour fragment was implanted intracranially in $B_6C_3F_1$ mice by trocar. Treatment was for 5 days.

Lewis lung carcinoma was propagated by s.c. implantation of an 8 $mm^3$ tumour fragment in the axillary region of $BDF_1$ mice. Treatment was given daily for 9 days p.o., i.p. or in the form of medicated diet. In the latter case, the animals were housed individually and food consumption was determined daily.

The Ehrlich ascites was studied in $CF_1$ mice. Tumour cells were adjusted to $6 \times 10^6$ and implanted i.p. Treatment was administered as described for the Lewis Lung tumour.

Sarcoma 180 (S180) was propagated in the ascitic form in $CF_1$ mice. Tumour cells were adjusted to $6 \times 10^6$ and implanted i.p. Experiments were also performed with the solid form of S180. A fragment of 40 mg was implanted s.c. into the dorsum. Unless otherwise indicated, S180 will refer to the ascitic form of this tumour.

Among the S180 and Ehrlich ascites recipients, there was a small percentage of tumour-free animals. Since they were equally distributed in the control and treated animals it was decided aebitrarily not to include these animals in the analyses. The animals were observed for 3 months or until death.

*Serum and ascites concentrations of lonidamine*

Concentrations of lonidamine in serum and ascites were determined according to the method of Catanese *et al.* (1978). The compound was extracted with n-heptane. Fluorimetric assay was performed using excitation and emission wavelengths of 305 and 345 nm respectively (uncorrected values). The sensitivity of the method was 1 $\mu g\,ml^{-1}$ serum or ascites. The identity of diclondazolic acid was checked by thin layer chromatography, using a method applied to AF 1312/TS (Burberi *et al.*, 1975).

In vivo *tests with heating*

Experiments were performed on S180- or Ehrlich ascites-bearing mice, implanted as described above. Heating was commenced 3 days following implantation of tumours essentially as described by Cioli & Silvestrini (1971). Mice were kept in an air-conditioned cabinet at $39 \pm 0.5$°C for 7 h a day for 4 days. Rectal temperature was checked daily 4 h after initiating the heating session. During each heating session lonidamine was administered immediately after optimization of body temperature. Controls and animals treated with lonidamine only were kept at $22 \pm 1$°C. Animals which died during heating or during the following 3 days were not considered, since their number was equally distributed among the various groups.

*Ultrastructural observations*

Ehrlich ascites-bearing mice were treated for 1 day only with either heating or lonidamine alone or with a combination of the two according to the above procedure. The Ehrlich ascites tumour cells were withdrawn at the end of heating. The controls were tumour cells from untreated animals.

The cells were fixed in PAF solution (Zamboni & De Martino, 1967) containing 2.5% of glutaraldehyde. After post-fixation in unbuffered osmium tetroxide the cells were dehydrated and embedded in Epon 812. This sections were stained with uranyl acetate and lead hydroxide and examined on a Siemens Elmiskop 101 electron microscope.

### Results

In vitro *tests*

Table I summarizes the effects of lonidamine, AF 1312/TS and reference compounds on KB, P-388 and HeLa cells.

Lonidamine produced no decrease in KB tumour cell growth in the concentration range from 1–10 $\mu g\,ml^{-1}$ but was effective at 100 $\mu g\,ml^{-1}$; 6-mercaptopurine, used as a positive control, was effective from 1 $\mu g\,ml^{-1}$.

The effects of lonidamine in the P-388 test were similar to those observed with the KB cells. MeCCNU, used as a positive control was effective at 10 $\mu g\,ml^{-1}$. In the HeLa test, lonidamine decreased tumour cell growth at 10 $\mu g\,ml^{-1}$. AF 1312/TS was active at concentrations 2.5 × greater than lonidamine, i.e. at 25 $\mu g\,ml^{-1}$.

In vitro *tests with heating*

Table II shows the effects of lonidamine and AF 1312/TS on survival of Chinese hamster cells (HA-1) heated at 43°C.

These results clearly show that in the presence of even small amounts of lonidamine, there was an

**Table I** Effects of lonidamine and reference drugs on different tumour systems *in vitro*

| *Tumour* | *Test substance* | *Concentration* ($\mu g\,ml^{-1}$) | *Per cent reduction of cell growth*[a] |
|---|---|---|---|
| KB | Lonidamine | 1 | 6.2 |
| | | 10 | 2.5 |
| | | 100 | 73.8 |
| | 6-mercapto-purine | 1 | 86.2 |
| | | 10 | 90.5 |
| | | 100 | 118.0 |
| P-388 | Lonidamine | 1 | 0.0 |
| | | 10 | 3.9 |
| | | 100 | 76.5 |
| | MeCCNU | 1 | 2.5 |
| | | 10 | 66.5 |
| | | 100 | 74.4 |
| HeLa | Lonidamine | 2.5 | 9.1 |
| | | 5 | 9.1 |
| | | 10 | 55.9 |
| | AF 1312/TS | 12.5 | 2.6 |
| | | 25 | 45.5 |
| | | 50 | 48.1 |
| | | 100 | 56.0 |

[a]% reduction calculated as final cell concentration of test group divided by 96 h (KB cells) or 48 h (P-388 and HeLa cells) control concentration.

appreciable increase in the cytotoxicity resulting from heat. According to results not reported in the Table, lonidamine *per se* did not affect plating efficiencies up to $50\,\mu g\,ml^{-1}$. Later experiments, carried out with the lysine salt of lonidamine and in the absence of DMSO showed much reduced hyperthermic sensitization. This suggests that DMSO facilitates entry of the drug into the cells. AF 1312/TS, up to the maximum concentration used ($50\,\mu g\,ml^{-1}$) did not increase the sensitivity of the cells to hyperthermia.

### In vivo *tests*

Lonidamine in a dose range $50–200\,mg\,kg^{-1}$ i.p. or $50–400\,mg\,kg^{-1}$ p.o. did not increase the life span of mice with P-388, L-1210, B-16 or ependymoblastoma tumours; at the highest doses used occasional toxic effects were observed. The lack of positive results on these tumours was demonstrated when lonidamine was administered to groups of 10–32 animals for each dose.

Table III summarizes the results of experiments conducted with the Lewis Lung tumour.

Anti-tumour activity was suggested in the experiments in which treatment was given i.p. In the first experiment, an increased life span of 32% was observed in the group treated with $50\,mg\,kg^{-1}$ i.p. In the second, the increase of life span was 92% at the dose, $50\,mg\,kg^{-1}$ i.p. and 106% at the dose,

**Table II** Effects of lonidamine and AF 1312/TS on survival of Chinese hamster cells (HA-1) heated at 43°C *in vitro*

| *Drug* | *Concentration* ($\mu g\,ml^{-1}$) | *DMSO* (*% of medium*) | *Duration of heat exposure* (*h*) | *Survival fraction* |
|---|---|---|---|---|
| Control | 0 | 0 | 2 | $10^{-1}$ |
| Lonidamine | 5 | 0.1 | 2 | $2\times10^{-2}$ |
| AF 1312/TS | 5 | 0.1 | 2 | $2\times10^{-1}$ |
| Control | 0 | 1.0 | 2 | $3\times10^{-2}$ |
| Lonidamine | 50 | 1.0 | 2 | $10^{-4}$ |
| AF 1312/TS | 50 | 1.0 | 2 | $4\times10^{-2}$ |
| Control | 0 | 0.1 | 3 | $5\times10^{-3}$ |
| Lonidamine | 5 | 0.1 | 3 | $6\times10^{-5}$ |
| AF 1312/TS | 5 | 0.1 | 3 | $3\times10^{-3}$ |
| Control | 0 | 1.0 | 3 | $5\times10^{-5}$ |
| Lonidamine | 50 | 1.0 | 3 | $<10^{-5}$ |
| AF 1312/TS | 50 | 1.0 | 3 | $3\times10^{-5}$ |

**Table III** Effects of lonidamine on Lewis Lung tumour in mice[a]

| *Daily dose (mg kg⁻¹) and route* | *MST[b] (day)* | *T/C%[c]* | *ILS%[d]* |
|---|---|---|---|
| 0 | 19.0 | | |
| 50 p.o. | 19.0 | 100 | |
| 100 p.o. | 16.3 | 86 | |
| 200 p.o. | 20.0 | 105 | 5 |
| 400 p.o. | 7.0 | 41 | |
| 0 | 16.1 | | |
| 50 i.p. | 21.3 | 132 | 32[f] |
| 100 i.p. | 19.0 | 118 | 18 |
| 0 | 17.3 | | |
| 50 i.p. | 33.3 | 192 | 92[g] |
| 100 i.p. | 35.8 | 206 | 106[g] |
| 0 | 32.0 | | |
| 100 medic. diet (0.08%)[e] | 31.3 | 97 | |

[a]10 mice were used for each dose.
[b]Median survival time.
[c]MST of test group/MST of 0 dose group × 100.
[d]Increased life span.
[e]Lonidamine concentration.
[f]$P < 0.05$.
[g]$P < 0.001$.

100 mg kg$^{-1}$ i.p. Administration by both gavage and medicated diet gave negative results.

Table IV summarizes the results of experiments performed with Ehrlich ascites.

Nine days treatment at 50 mg kg$^{-1}$ p.o. daily produced a 25% increase in life span which was not significant; no effect was observed with 100 mg kg$^{-1}$ p.o. daily. Fifteen days treatment at 100 mg kg$^{-1}$ p.o. daily, decreased the life span. Lonidamine was inactive when given in the medicated diet.

Table 5 summarizes the results of experiments performed with S180.

A 22% increase of life span was observed with 25 mg kg$^{-1}$ p.o. of lonidamine. This value was lower than the one considered indicative of anti-tumour action (Geran *et al.*, 1972) and was not statistically significant. This was supported by the lack of effect at 50 and 100 mg kg$^{-1}$ p.o. These doses are much lower than those producing general toxicity (see Table III).

Administration of lonidamine in the diet resulted in a dose-related increase in life span. AF 1312/TS was as active as lonidamine at 10× the dose, i.e. 1250 mg kg$^{-1}$ compared with 125 mg kg$^{-1}$ lonidamine in the diet. Using the S180 in the solid form the efficacy of lonidamine was confirmed.

### *Serum and ascites concentrations of lonidamine*

Lonidamine was measured in the serum of normal and S180-bearing animals following exposures of different duration via the diet. In tumour-bearers,

**Table IV** Effects of lonidamine on the Ehrlich ascites tumour in mice

| *Daily dose (mg kg⁻¹) and route* | *No. mice* | *Days of treatment* | *MST[a] (day)* | *T/C%[b]* | *ILS%[c]* |
|---|---|---|---|---|---|
| 0 | 19 | 1–9 | 23.2 | | |
| 25 p.o. | 20 | 1–9 | 25.3 | 109 | 9 |
| 50 p.o. | 18 | 1–9 | 29.2 | 125 | 25 |
| 100 p.o. | 18 | 1–9 | 22.4 | 96 | |
| 0 | 10 | 1–15 | 19.2 | | |
| 100 p.o. | 10 | 1–15 | 12.2 | 64 | |
| 0 | 18 | | 19.4 | | |
| 250 medic diet (0.17%)[d] | 19 | 0–death | 21.2 | 109 | 9 |
| 0 | 28 | | 22.4 | | |
| 600 medic. diet (0.33%) | 29 | 0–6 | 18.4 | 82.3 | |

[a]Median survival time.
[b]MST of test group/MST of 0 dose group × 100.
[c]Increased life span.
[d]Lonidamine concentration in parentheses.

**Table V** Effects of lonidamine and AF 1312/TS on S180 in mice. Unless otherwise indicated the tumour was in the ascitic form

| *Test Substance* | *Daily dose (mg kg$^{-1}$) and route* | *No. mice* | *Days of treatment* | *MST*[a] *(day)* | *T/C%*[b] | *ILS %*[c] |
|---|---|---|---|---|---|---|
| Lonidamine | 0 | 19 | 1–9 | 22.4 | | |
| | 25 p.o. | 20 | 1–9 | 27.4 | 122 | 22 |
| | 50 p.o. | 15 | 1–9 | 22.0 | 98 | |
| | 100 p.o. | 19 | 1–9 | 24.4 | 109 | 9 |
| | 0 | 20 | | 20.3 | | |
| | 62 medic. diet (0.04%)[e] | 19 | 0–death | 21.1 | 104 | 4 |
| | 0 | 37 | | 21.2 | | |
| | 125 medic. diet (0.00%) | 37 | 0–death | 26.3 | 124 | 24[f] |
| | 0 | 30 | | 23.3 | | |
| | 250 medic. diet (0.17%) | 29 | 0–death | 35.3 | 151 | 51[g] |
| | 0 | 21 | | 54.5[d] | | |
| | 125 medic. diet (0.08%) | 19 | 0–death | 72.5[d] | 133 | 33[h] |
| AF 1312/TS | 0 | 30 | | 23.3 | | |
| | 1250 medic. diet (0.8%) | 30 | 0–death | 29.3 | 126 | 26[f] |

[a]Median survival time. [b]MST of test group/MST of 0 dose group × 100. [c]Increased life span. [d]S180 in the solid form. [e]Lonidamine concentration in parentheses. [f]$P<0.01$. [g]$P<0.001$. [h]$P<0.05$.

treatment was commenced concomitantly with tumour implantation. The daily doses of 125 and 250 mg kg$^{-1}$ correspond to those which in the previous experiments produced a significant increase in life span. The results are summarized in Table VI.

In tumour bearers, these doses produced serum concentrations in the range of 12–22 and 18–28 $\mu$g ml$^{-1}$ respectively; similar concentrations were found in the ascitic fluid. During the first week of treatment the serum concentrations were higher in normal animals than in tumour-bearers; no appreciable difference was observed the following week. Body fluid levels of lonidamine were also estimated in mice with Ehrlich ascites which, in the previously described experiments, proved to be resistant to lonidamine. The drug was given in the diet at a daily dose of 250 mg kg$^{-1}$ (0.17%). The animals were killed and examined after 7 days of treatment. The concentrations were $17.9 \pm 1.61$ and $22.3 \pm 2.14$ $\mu$g ml$^{-1}$ in the serum and ascitic fluid respectively.

### In vivo *tests with heating*

Table VII summarizes the effects of lonidamine and heating, both alone and together, on S180 and Ehrlich ascites in mice. Treatments were performed daily on days 3–6 following tumour implantation.

Lonidamine alone at the daily dose of 50 mg kg$^{-1}$ p.o., had no effect on S180. Combination with heat resulted in a 29% increase in life span.

Lonidamine at the daily dose of 25 or 50 mg kg$^{-1}$ p.o. did not significantly increase the life span of

**Table VI** Concentrations of lonidamine in the serum of normal and S180-bearing mice following dietary administration

| Animals | *Daily dose*[a] *(mg kg$^{-1}$)* | *Fluid examined* | *µg ml$^{-1}$ on days* 3 | 6 | 7 | 12 | 14 |
|---|---|---|---|---|---|---|---|
| Normal mice | 62 | serum | 16.7 ±1.17 | 12.1 ±0.51 | | 14.1 ±0.13 | |
| | 125 | serum | 32.5 ±2.02 | 23.1 ±2.59 | | 20.2 ±3.15 | |
| S180—bearing mice | 125 | serum | 22.3 ±1.48 | | 12.2 ±0.76 | | 20.7 ±3.05 |
| | | ascites | | | 11.3 ±1.32 | | 17.9[b] |
| | 250 | serum | 27.8 ±1.45 | | 18.1 ±2.53 | | 19.4 ±1.56 |
| | | ascites | | | 21.1 ±2.85 | | 19.9 ±0.85 |

[a]The doses given correspond to drug concentrations of 0.04, 0.08 and 0.17%, respectively. For each time and dose level 3 or more animals were used. [b]Studied in one mouse only.

**Table VII** Effects of lonidamine and heating (39°C) on S180 and Ehrlich ascites tumour in mice

| *Tumour* | *Treatment*[a] | *Daily dose of lonidamine (mg kg$^{-1}$ p.o.)* | *No. mice* | *Body temp.*[b] *(°C; mean ± se)* | *MST*[c] *(day)* | *T/C%*[d] | *ILS*[e] |
|---|---|---|---|---|---|---|---|
| S180 | control | 0 | 16 | 37.8±0.18 | 24.3 | | |
| | lonidamine | 50 | 15 | 38.0±0.13 | 24.1 | 99 | |
| | heating | 0 | 12 | 39.6±0.15 | 26.3 | 109 | 9 |
| | heating + lonidamine | 50 | 17 | 39.4±0.08 | 31.3 | 129 | 29[f] |
| Ehrlich ascites | control | | 16 | 37.0±0.26 | 18.3 | | |
| | lonidamine | 25 | 15 | 36.9±0.18 | 19.9 | 109 | 9 |
| | heating | | 16 | 39.0±0.12 | 20.4 | 111 | 11 |
| | heating + lonidamine | 25 | 18 | 39.0±0.12 | 23.2 | 127 | 27[f]) |
| | control | | 15 | 36.9±0.20 | 20.2 | | |
| | lonidamine | 50 | 18 | 37.2±0.16 | 22.2 | 110 | 10 |
| | heating | | 17 | 39.1±0.10 | 23.3 | 115 | 15 |
| | heating + lonidamine | 50 | 17 | 39.0±0.12 | 27.4 | 135 | 35[g] |

[a]On days 3–6; [b]Average of 4 daily determinations; [c]Median survival time; [d]MST of test group/MST of 0 dose group × 100; [e]Increased Life Span; [f]$P<0.05$; [g]$P<0.001$.

animals bearing Ehrlich ascites. Heating was likewise without any significant effect. Together the 2 treatments resulted in an increase of life span of 27 and 35%, at doses of 25 and 50 mg kg$^{-1}$ p.o. lonidamine respectively.

*Ultrastructural observations*

Two main types of Ehrlich ascites tumour cells were visible in the untreated controls. The first type was more numerous and characterized by mitochondria with lamellar cristae (Figure 1), regularly arranged (orthodox form); the second type revealed mitochondria with dense matrix and expanded intercristal space (Figure 2) or condensed-like form (De Martino *et al.*, 1981). In the animals treated with lonidamine only (50 mg kg$^{-1}$ p.o.) there was no increase in the number of ascitic cells of the second type, but heating increased their number. In many cells, several mitochondria assume an unusual form of condensation (Figure 3). The combination of lonidamine (50 mg kg$^{-1}$ p.o.) with heating induced severe mitochondrial damage such as rarefaction of the matrix and disruption of the cristae in numerous tumour cells (Figure 4).

### Discussion

These experiments provide data on three issues which will be discussed separately: (i) the narrow spectrum of activity of lonidamine in the battery of tests currently used for the screening of anti-tumour agents; (ii) its anti-tumour activity in combination with hyperthermia; (iii) the blood levels corresponding to anti-tumour activity in a specific bioassay relative to those found in man (Besner *et al.*, 1981 unpublished).

(i) The tumour systems currently used for the screening of anti-tumour agents differ sharply from naturally-occurring neoplasms; not only are they based upon transplantable tumours, but the tumour–host relationship (which seemingly influences the course of natural diseases) is altered. Responsiveness to currently available anti-tumour agents has been the principal, if not the only, criterion for the choice of these systems (Gellhorn & Hirschberg, 1955; Goldin *et al.*, 1966; Wood, 1977). There are however, some evident limitations attached to this policy of screening anti-tumour agents. Most of the presently available agents are similar in their basic mechanism of action, *viz.*, they are anti-mitotics. Consequently, screening focuses on a specialized type of anti-tumour activity, and there is no evidence that it will eventually identify agents which act by different mechanisms. Such might be the case with lonidamine. All the available data indicate that this drug primarily affects the energy metabolism (and not the replicative process) of the cancer cell and this should be taken into

**Figure 1** Detail of cytoplasm of Ehrlich ascites tumour cell from untreated animal showing typical orthodox mitochondria with lamellar cristae (×35,000).

**Figure 2** Detail of cytoplasm of Ehrlich ascites tumour cell from untreated animal showing mitochondria with dense matrix and expanded intercristal space (condensed form) ( × 35,000).

**Figure 3** Tumour cells from heated animals (duration of treatment, 1 day). In several cells mitochondria are extremely condensed. Some show unusual arrangement of the cristae and matrix (arrowed) ( × 20,000).

**Figure 4** Tumour cells from animals which received lonidamine (50 mg kg$^{-1}$ p.o.) plus heat treatment (1 day only). The mitochondria of some cells appear swollen with loss of the matrix and derangement of the cristae ( × 18,000).

account when interpreting the results of this study. Thus, they do not necessarily indicate that lonidamine is a narrow spectrum anti-tumour agent, but rather that current experimental tumour models are unsuitable for the study of drugs acting on the energy metabolism of the cell.

(ii) The study of hyperthermia relates to both the problem of the mechanism of action of lonidamine and its potential therapeutic interest in combination with hyperthermia. As stated in the **Introduction**, previous studies indicate that the condensed mitochondrion is the target for the activity of lonidamine (Floridi *et al.*, 1980; 1981; Silvestrini, 1981). Hyperthermia was studied on the basis of the speculation that it could produce an imbalance between energy requirements and the oxygen supply of the tumour cells, thus leading to the formation of condensed mitochondria. This has been corroborated here by ultrastructural observations showing the appearance of condensed mitochondria under the influence of hyperthermia. The finding that a degree of hyperthermia, inactive alone, has significant anti-tumour activity when combined with lonidamine is in agreement with the working hypothesis that the state of the energy metabolism of the cell is the critical factor for the activity of lonidamine, but is not conclusive. The question whether hyperthermia reproduces a metabolic state in experimental tumours which also occurs in some stages of naturally-occurring tumours is of practical importance, but cannot be answered by data presently available. Apart from these theoretical considerations, our data suggest a potential use of the lonidamine–hyperthermia combination in the treatment of cancer. A recent study (Kim *et al.*, 1982) has confirmed that lonidamine potentiates the anti-tumour activity of hyperthermia both *in vivo* and *in vitro*, showing that *in vitro* pH represents a critical factor for this phenomenon.

(iii) The doses of lonidamine active on S180 have been shown to correspond to blood concentrations in the range of 12–28 $\mu g\,ml^{-1}$; since these blood levels are in the range of those detected in patients treated with lonidamine (Besner *et al.*, 1981 unpublished), these results further stress the potential of lonidamine for the treatment of cancer.

### References

BURBERI, S., CATANESE, B., CIOLI, V. SCORZA BARCELLONA, P. & SILVESTRINI, B. (1975). Antispermatogenic activity of 1-p-chlorobenzyl-1H-3-carboxylic acid (AF 1312/TS) in rats. II. A study of treatments of duration between 5 and 180 days. *Exp. Molec. Pathol.*, **23**, 308.

CAPUTO, A. (1981). Preliminary results in some tumour systems with indazolecarboxylic acids. Chemotherapy **27** (Suppl. 2), 107.

CATANESE, B., CIOLI, V., DE MARTINO, C. & SILVESTRINI, B. (1978). A comparative study of serum and testicular concentrations of AF 1312/TS or 1-4-chlorophenyl-methyl-1H-indazole-3-carboxylic acid and diclondazolic acid or 1,2-4-dichlorophenyl-methyl-1H-indazole-3-carboxylic acid. *Pharmacol. Res. Commun.*, **10**, 261.

CIOLI, V. & SILVESTRINI, B. (1971). Comparative effects of heating and fasting in mice, with particular reference to development of Sarcoma 180. *Br. J. Cancer*, **25**, 149.

CORSI, G., PALAZZO, G., GERMANI, C., SCORZA BARCELLONA, P. & SILVESTRINI, B. (1976). 1-halobenzyl-1H-indazole-3-carboxylic acids. A new class of antispermatogenic agents. *J. Med. Chem.*, **19**, 778.

DE MARTINO, C., FLORIDI, A., MARCANTE, M.L. & 4 others (1979). Morphological, histochemical and biochemical studies on germ cell mitochondria of normal rats. *Cell. Tiss. Res.*, **196**, 1.

DE MARTINO, C., MALORNI, W., BELLOCCI, M., FLORIDI, A. & MARCANTE, M.L. (1981). Effects of AF 1312/TS and lonidamine on mammalian testis. A morphological study. *Chemotherapy*, **27**, (Suppl. 2), 27.

FAWCETT, D.W. (1970). A comparative view of sperm ultrastructure. *Biol. Reprod.*, **2**, 90.

FLORIDI, A., DE MARTINO, C., MARCANTE, M.L., SCORZA BARCELLONA, P. & SILVESTRINI, B. (1980). Morphological and biochemical modifications of rat germ cell mitochondria induced by new antispermatogemic compounds. *Ultramicroscopy*, **5**, 363.

FLORIDI, A., BELLOCCI, M., PAGGI, M.G., MARCANTE, M.L. & DE MARTINO, C. (1981). Changes of energy metabolism in the germ cells and Ehrlich ascites tumour cells. *Chemotherapy*, **27**, (Suppl. 2), 50.

FLORIDI, A., PAGGI, M.G., MARCANTE, M.L., SILVESTRINI, B., CAPUTO, A. & DE MARTINO, C. (1981a). Lonidamine, a selective inhibitor of aerobic glycolysis of murine tumour cells. *J. Natl. Cancer Inst.*, **66**, 497.

GELLHORN, A. & HIRSCHBERG, E. (1955). Investigation on diverse systems for cancer chemotherapy screening. *Cancer Res.*, **15**, (Suppl. 3), 1.

GERAN, R.I., GREENBERG, N.H. & MCDONALD, M.M. *et al.* (1972). Protocols for screening chemical agents and natural products against animal tumours and other biological systems. *Cancer Chemother. Rep.*, (Part 3), **3**, 1.

GOLDIN, A., SERPICK, A.A. & MANTEL, N. (1966). A commentary. Experimental screening procedures and clinical predictability value. *Cancer Chemother. Rep.*, **50**, 173.

HACKENBROCK, C.R. (1966). Ultrastructural bases for metabolically linked mechanical activity in mitochondria. I. Reversible ultrastructural changes with changes in metabolic steady state. *J. Cell Biol.*, **30**, 269.

HACKENBROCK, C.R. (1968). Ultrastructural bases for metabolically linked mechanical activity in mitochondria. II. Electron transport linked ultrastructural transformation in mitochondria. *J. Cell Biol.*, **37,** 345.

HACKENBROCK, C.R., REHN, T.G., WEINBACH, E.C. & LEMASTERS, J.J. (1971). Oxidative phosphorylation and ultrastructural transformation in mitochondria in the intact ascites tumour cell. *J. Cell. Biol.*, **51,** 123.

HEYWOOD, R., JAMES, R.W., SCORZA BARCELLONA, P., CAMPANA, A. & CIOLI, V. (1981). Toxicological studies on 1-substituted-indazole-3-carboxylic acids. *Chemotherapy*, **27,** (Suppl. 2), 91.

KIM, J.H. *et al.* (1982). Hyperthermia potentiation by lonidamine. *Proceedings of 73rd Annual Meeting of the American Association for Cancer Research* (April 1982) Vol. **23,** 194.

MACHADO, DE DOMENECHI, E., DOMENECH, C.E., AOKI, A. & BLANCO, A. (1972). Association of the testicular lactate dehydrogenase isozyme with a special type of mitochondria. *Biol. Reprod.*, **6,** 136.

PUCK, T.T. & MARCUS, P.T. (1956). Action of x-rays on mammalian cells. *J. Exp. Med.*, **103,** 653.

SCORZA BARCELLONA, P., CAMPANA, A., DE MARTINO, C. & SILVESTRINI, B. (1982). The embryotoxicity of a new class of antispermatogenic agents: the indazole-1H-carboxylic acids. *Arch. Toxicol.*, (Suppl. 5) 197.

SILVESTRINI. B., DE MARTINO, C., CIOLI, V., CAMPANA, A., MALORNI, W. & SCORZA BARCELLONA, P. (1978). Antispermatogenic activity of diclondazolic acid in rats. *Recent Prog. Androl.*, (L'Aquila) **14,** 453.

SILVESTRINI, B. (1981). Basic and applied research in the study of indazole carboxylic acids. *Chemotherapy*, **27,** (Suppl. 2), 9.

WOOD, H.B. (1977). Selection of agents for the tumour screen of potential new antineoplastic drugs. In: *U.S.A.-U.S.S.R. Monograph—Methods of Development of new Anticancer Drugs—Natl. Cancer Inst. Monog.*, **45,** 15.

ZAMBONI, L. & DE MARTINO, C. (1967). Buffered picric acid-formaldehyde: A new rapid fixative for electron microscopy. *J. Cell. Biol.*, **35,** 148a.