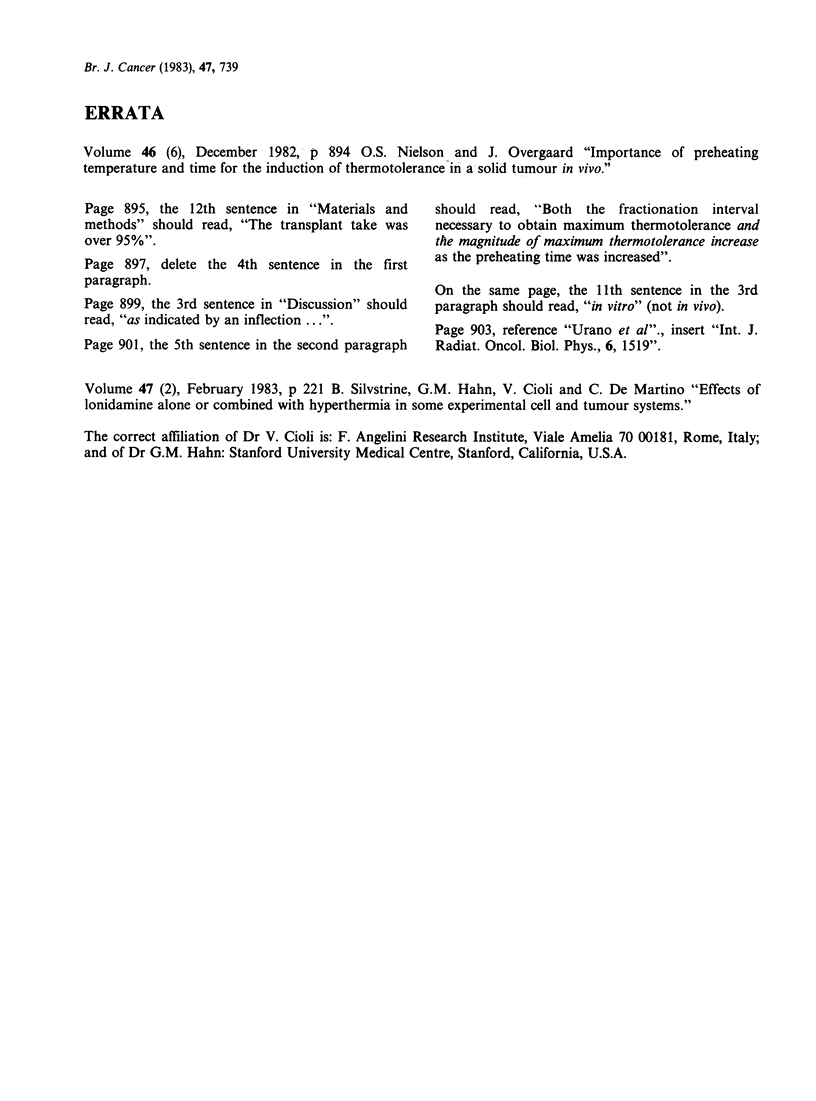# Errata

**Published:** 1983-05

**Authors:** 


Volume **47** (2), February 1983, p 221 B. Silvstrine, G.M. Hahn, V. Cioli and C. De Martino "Effects of lonidamine alone or combined with hyperthermia in some experimental cell and tumour systems."

The correct affiliation of Dr V. Cioli is: F. Angelini Research Institute, Viale Amelia 70 00181, Rome, Italy; and of Dr G.M. Hahn: Stanford University Medical Centre, Stanford, California, U.S.A.